# Neoadjuvant Dual Checkpoint Inhibitors vs Anti-PD1 Therapy in High-Risk Resectable Melanoma: A Pooled Analysis

**DOI:** 10.1001/jamaoncol.2023.7333

**Published:** 2024-03-28

**Authors:** Ankit Mangla, Chanmi Lee, Matthew M. Mirsky, Margaret Wang, Luke D. Rothermel, Richard Hoehn, Jeremy S. Bordeaux, Bryan T. Carroll, Jason Theuner, Shawn Li, Pingfu Fu, John M. Kirkwood

**Affiliations:** 1 Department of Hematology and Oncology, University Hospitals Seidman Cancer Center, Cleveland, Ohio; 2 Department of Hematology and Oncology, Case Western Reserve University School of Medicine, Cleveland, Ohio; 3 Case Comprehensive Cancer Center, Cleveland, Ohio; 4 Department of Internal Medicine, University Hospitals Cleveland Medical Center, Cleveland, Ohio; 5 Department of Surgical Oncology, University Hospitals Cleveland Medical Center, Cleveland, Ohio; 6 Department of Dermatology, University Hospitals Cleveland Medical Center, Cleveland, Ohio; 7 Department of Otolaryngology, University Hospitals Cleveland Medical Center, Cleveland Ohio; 8 Department of Population and Quantitative Health Sciences, Case Western Reserve University School of Medicine, Cleveland, Ohio; 9 Department of Medicine and Dermatology, UPMC Hillman Cancer Center and Melanoma and Skin Cancer Program, University of Pittsburgh School of Medicine, Pittsburgh, Pennsylvania

## Abstract

**Importance:**

Despite the clear potential benefits of neoadjuvant therapy, the optimal neoadjuvant regimen for patients with high-risk resectable melanoma (HRRM) is not known.

**Objective:**

To compare the safety and efficacy of dual checkpoint inhibitors with anti–programmed cell death protein-1 (anti-PD1) therapy in a neoadjuvant setting among patients with HRRM.

**Design, Setting, and Participants:**

In this pooled analysis of clinical trials, studies were selected provided they investigated immune checkpoint inhibitor treatment, were published between January 2018 and March 2023, and were phase 1, 2, or 3 clinical trials. Participant data included in the analysis were derived from trials evaluating the efficacy and safety of anti-PD1 monotherapy and the combination of anti–cytotoxic T lymphocyte-associated protein-4 with anti-PD1 in the neoadjuvant setting, specifically among patients with HRRM.

**Interventions:**

Patients were treated with either anti-PD1 monotherapy; dual checkpoint inhibition (DCPI) with a conventional dose of 3-mg/kg ipilimumab and 1-mg/kg nivolumab; or DCPI with an alternative-dose regimen of 1-mg/kg ipilimumab and 3-mg/kg nivolumab.

**Main Outcomes and Measures:**

The main outcomes were radiologic complete response (rCR), radiologic overall objective response (rOOR), and radiologic progressive disease. Also, pathologic complete response (pCR), the proportion of patients undergoing surgical resection, and occurrence of grade 3 or 4 immune-related adverse events (irAEs) were considered.

**Results:**

Among 573 patients enrolled in 6 clinical trials, neoadjuvant therapy with DCPI was associated with higher odds of achieving pCR compared with anti-PD1 monotherapy (odds ratio [OR], 3.16; *P* < .001). DCPI was associated with higher odds of grade 3 or 4 irAEs compared with anti-PD1 monotherapy (OR, 3.75; *P* < .001). When comparing the alternative-dose ipilimumab and nivolumab (IPI-NIVO) regimen with conventional-dose IPI-NIVO, no statistically significant difference in rCR, rOOR, radiologic progressive disease, or pCR was noted. However, the conventional-dose IPI-NIVO regimen was associated with increased grade 3 or 4 irAEs (OR, 4.76; *P* < .001). Conventional-dose IPI-NIVO was associated with greater odds of achieving improved rOOR (OR, 1.95; *P* = .046) and pCR (OR, 2.99; *P* < .001) compared with anti-PD1 monotherapy. The alternative dose of IPI-NIVO also was associated with higher odds of achieving rCR (OR, 2.55; *P* = .03) and pCR (OR, 3.87; *P* < .001) compared with anti-PD1 monotherapy. The risk for grade 3 or 4 irAEs is higher with both the conventional-dose (OR, 9.59; *P* < .001) and alternative-dose IPI-NIVO regimens (OR, 2.02; *P* = .02) compared with anti-PD1 monotherapy.

**Conclusion and Relevance:**

In this pooled analysis of 6 clinical trials, although DCPI was associated with increased likelihood of achieving pathological and radiologic responses, the associated risk for grade 3 or 4 irAEs was significantly lower with anti-PD1 monotherapy in the neoadjuvant setting for HRRM. Additionally, compared with alternative-dose IPI-NIVO, the conventional dose of IPI-NIVO was associated with increased risk for grade 3 or 4 irAEs, with no significant distinctions in radiologic or pathologic efficacy.

## Key Points

**Question:**

What is the optimal neoadjuvant regimen for patients with operable node-positive, high-risk stage III and IV melanoma?

**Findings:**

In this pooled analysis of 573 patients from 6 clinical trials, the risk for adverse events was lower with anti–programmed cell death protein-1 agents in the neoadjuvant setting. However, dual checkpoint inhibition was associated with an increased likelihood of attaining a complete pathologic and radiologic response.

**Meaning:**

This study suggests that although attaining a favorable pathologic and radiologic response was more likely with neoadjuvant dual checkpoint inhibitor therapy, the occurrence of a grade 3 or 4 adverse event was also higher.

## Teaser

This pooled analysis of 6 clinical trials describes the safety and efficacy of different courses of immune checkpoint inhibitor treatment in a neoadjuvant setting among patients with high-risk resectable melanoma.

## Introduction

Immune checkpoint inhibitors (ICIs) have become the standard of care in the treatment of patients with advanced melanoma.^1^ All patients with clinical stage III and stage IV high-risk resectable melanoma (HRRM) are candidates for adjuvant systemic therapy with ICIs or with BRAF/MEK inhibitors (v-raf murine sarcoma viral oncogene homolog B and mitogen-activated protein kinase inhibitors) if the tumor of a given patient carries the *BRAF* V600E or V600K sequence variations. The premise of adjuvant therapy has been that treatment of distant micrometastatic tumor deposits may improve the cure of this disease after standard surgery.^2^ Adjuvant therapy trials in melanoma have shown benefits since 1996, demonstrating a reduction in the likelihood of recurrence and/or death compared with observation or placebo groups expressed in hazard ratios ranging from 0.42 to 0.81.^3,4,5,6,7^ However, in patients with clinical stage III melanoma (macrometastasis to lymph nodes), the relapse rate is as high as 40% within the first 2 years, even with adjuvant immunotherapy.^3,5^

The neoadjuvant approach to the management of patients with HRRM was first pursued with interferons in 2006 and has gained traction in the past decade.^8^ It is hypothesized that neoadjuvant therapy (NAT) with ICIs leads to a better immune response due to the delivery of immunomodulators when the tumor is still present in measurable (generally nodal) resectable disease.^9^ This, in turn, may lead to increased peripheral T-cell receptor clonal diversity, which may reflect the proliferation and maturation of intratumoral T-cell populations.^10^ Uniquely, NAT allows for the assessment of the pathologic response of tumors, which not only provides information about the immunologic activity of the various interventions but can also have a congruent effect on relapse-free survival, distant metastasis-free survival, and overall survival.^11,12^ In this pooled analysis, we have collected data from the published trials of neoadjuvant ICI treatment for patients with HRRM and analyzed them for safety and efficacy.

## Methods

In this pooled analysis, we have collected data from the published trials of neoadjuvant ICI treatment for patients with HRRM and analyzed them for safety and efficacy. Adherence to the Preferred Reporting Items for Systematic Reviews and Meta-analyses (PRISMA) reporting guideline was ensured. This study was exempt from institutional review board approval and informed consent because patient data were deidentified.

### Definitions

We defined outcomes based on the definitions used in the clinical trials (eTable 1 in Supplement 1). For this pooled analysis, dual checkpoint inhibition (DCPI) refers to the combined regimen of ipilimumab and nivolumab (IPI-NIVO) only. Patients treated with anti–lymphocyte activation gene-3 combination therapy, consisting of a fixed-dose combination of nivolumab and relatlimab (Opdualag [Bristol Myers Squibb]), were not included in the pooled analysis because of small sample sizes, and the heterogeneity of the treatment groups was reduced. Conventional-dose IPI-NIVO refers to the combination of 3-mg/kg ipilimumab and 1-mg/kg nivolumab, and alternative dose IPI-NIVO refers to 1-mg/kg ipilimumab and 3-mg/kg nivolumab.

### Literature Sources and Search Strategy

For published literature, the PubMed database was searched with the keywords “neoadjuvant AND melanoma” and a date range of January 2018 to March 2023, yielding 393 publications. The search was verified with the keywords “(neoadjuvant therapy[mesh] OR neoadjuvant*[tiab] OR preoperative therapy*[tiab]) AND (melanoma[mesh] OR melanoma*[tiab]) AND (clinical trial[publication type] OR trial*[ti] OR random*[ti]) AND English[lang]” which yielded 58 search results. The 2 lists were compared for overlaps and underwent screening, as described in the next section.

### Study Selection

Eligible studies included prospective phase 1, 2, and 3 randomized clinical trials, single-arm trials, pooled analyses, and planned future trials investigating the efficacy of NAT for patients with HRRM. Abstracts were screened by title for relevance, and studies that did not include immunotherapies as neoadjuvant interventions were excluded. The studies that appeared in multiple publications with more recent data were excluded after the index (initial) report. Studies evaluating interferons, oncolytic viruses, and vaccines were excluded. Two individuals, C.L. and M.M.M., scanned titles and abstracts of all studies for initial selection, and the selected abstracts were reviewed by several authors (A.M., C.L., M.M.M., M.W., and J.M.K.). Discrepancies were resolved by consensus (Figure).

**Figure.**
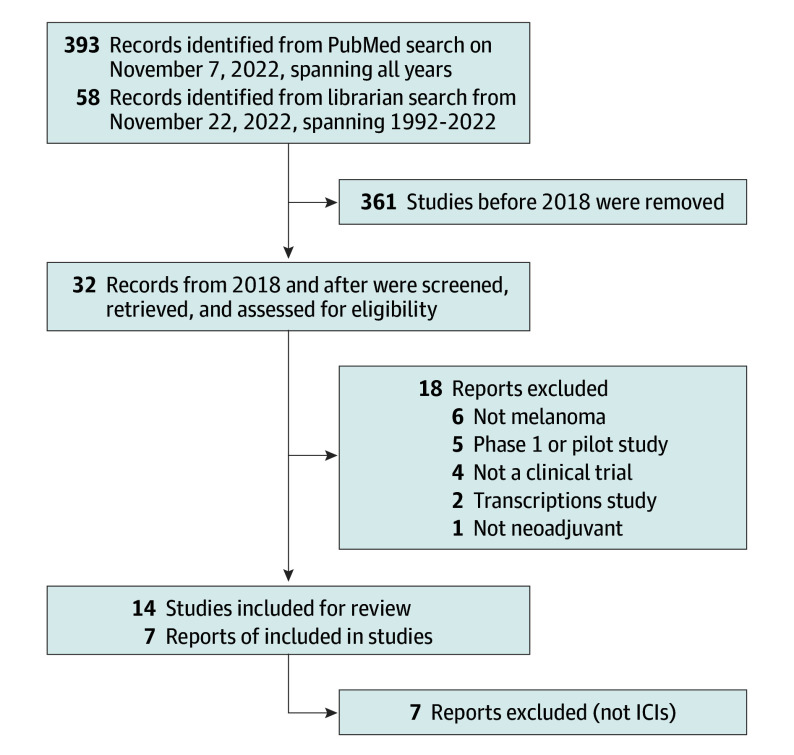
Identification of Studies via Databases and Registers This figure displays the PRISMA flowchart for recruitment strategy. ICI indicates immune checkpoint inhibitor.

### Statistical Analysis

For summary data analysis, studies were grouped based on treatment type (anti–programmed cell death protein-1 [anti-PD1], conventional-dose IPI-NIVO therapy, or alternative-dose IPI-NIVO therapy). The detailed analysis included data derived only from full published articles. The confounding factors were not adjusted due to lack of access to individual patient data. The difference of binary outcomes (eg, various response data and grade 3 or 4 immune-related adverse events [irAEs]) between the 2 treatment types was compared using the *Z* test (a variant of the χ^2^ test) with the effect size of treatment estimated using odds ratios (ORs) and corresponding 95% CIs, with *P* < .05 set as statistically significant (eMethods in Supplement 1).

## Results

A total of 573 patients were treated with ICIs in the 6 clinical trials that were included in this analysis. Four trials used DCPI with a combination of anti–cytotoxic T lymphocyte-associated protein-4 (anti-CTLA-4) and anti-PD1 therapy. Additionally, 2 trials exclusively used anti-PD1, and another trial involved the use of anti–lymphocyte activation gene-3 in combination with anti-PD1. The majority of patients had nonacral cutaneous melanoma, although exact numbers could not be ascertained due to incomplete reporting. All patients were naive to ICIs. End points varied among trials depending on design (Table 1).^10,13,14,15,16,17,18^

**Table 1.** Descriptions of Studies of Neoadjuvant Immune Checkpoint Inhibitors in Patients With High-Risk Resectable Melanoma

Study	Design	Drugs	Patients, No.	Melanoma stage	Melanoma type	Treatment-naive patients, No.^a^	End points	Key clinical observations
Amaria et al,^13^ 2018	Randomized, phase 2	Group 1: nivolumab, 3 mg/kg every 14 d (4 doses) Group 2: ipilimumab, 3 mg/kg + nivolumab, 1 mg/kg every 21 d (3 doses) All patients receive 13 doses of adjuvant nivolumab	23	IIIB, IIIC, IIID, and IV	Cutaneous only; no mucosal or acral melanoma	19	Response rates; trAE; immune correlate of responses	Improved RFS, DMFS, and OS noted in patients with pCR Improved RFS, PFS, DMFS, and OS with DCPI (not statistically significant)
Blank et al,^10^ 2018	Randomized feasibility study, phase 1B	Group 1: ipilimumab 3 mg/kg + nivolumab 1 mg/kg (every 3 wk); adjuvant treatment (4 cycles after surgery) Group 2: neoadjuvant treatment (2 cycles before surgery and 2 cycles after surgery)	20	IIIB and IIIC	Cutaneous	20	Primary: feasibility, safety, immune-activating capability Secondary: trAE; response; RFS, correlatives	No relapse in those who had pathologic response Relapse rate of 80% within the first 2 y RFS and OS may be better with neoadjuvant DCPI
Huang et al,^14^ 2019	Single arm, phase 1B	Pembrolizumab, 200-mg single dose, in neoadjuvant setting; surgery; and adjuvant therapy with pembrolizumab	29	Clinical stage III, resectable stage IV	Cutaneous; excluded mucosal or uveal	29	Response trAE; biomarkers	Major pathologic response correlates with 100% DFS at 2 y T-reg proliferation was associated with poor RFS
Rozeman et al,^15^ 2019	Randomized clinical trial, phase 2	Group 1: ipilimumab, 3 mg/kg + nivolumab 1 mg/kg every 21 d for 2 cycles Group 2: ipilimumab, 1 mg/kg + nivolumab, 3 mg/kg, every 21 d for 2 cycles Group 3: ipilimumab, 3 mg/kg every 3 wk for 2 cycles followed by nivolumab every 2 wk for 2 cycles	89	Stage III	NR	NR	trAE within first 12 wk; radiologic response at 6 wk; pathologic response at 6 wk	2-y RFS was significantly higher for those with a pathologic response Low TMB and low IFN-γ are associated with the lowest survival and pathologic response rate High TMB + high IFN-γ were associated with the best pathologic response and EFS rate
Amaria et al,^16^ 2022	Single arm	Nivolumab, 480 mg + relatlimab, 160 mg intravenously 4 wk for 2 doses; surgery; and 10 doses of nivolumab, 480 mg + relatlimab, 160 mg, every 4 wk	30	IIIB to IIID, M1a (completely resected)	NR	29 (1 Patient received BRAF/MEK inhibitors before this study)	Primary: pCR Secondary: trAE/safety; response rate; correlative studies	1-y and 2-y RFS was significantly higher in patients with pathologic response Increase in immune cell infiltration and decrease in M2 macrophage were associated with higher pathologic response
Reijers et al,^17^ 2022	Single arm	Ipilimumab, 1 mg/kg, and nivolumab, 3 mg/kg for 3 wk, 2 cycles Pathologic assessment of ILND, TLND, and adjuvant therapy based on pathologic response	99	Stage III with clinically palpable nodes	NR	NR	Primary: RFS at 24 mo; rate of pathologic pCR, pnCR, pNR in the ILND, radiologic response, toxic effects Secondary: long-term RFS; correlative studies	MPR in ILND was associated with better RFS and DMFS ILND, TLND, and adjuvant therapy had better patient outcomes than no therapy TLND omission improved reduced surgical morbidity and better HRQoL
Patel et al,^18^ 2023	Randomized clinical trial, phase 2	Group 1: pembrolizumab, 200 mg, 3 wk for 3 doses before surgery, then surgery, followed by 14 more doses Group 2: surgery, followed by pembrolizumab, 200 mg, 3 wk for 17 cycles	313	Stage IIIB to IIID, and IV (completely resected)	Cutaneous (296 patients); mucosal (4 patients); and acral (9 patients)	NR	Primary: EFS Secondary: trAE; response rates; surgical resection	EFS was significantly better in the neoadjuvant group

Abbreviations: DCPI, dual checkpoint inhibition; DFS, disease-free survival; DMFS, distant metastasis-free survival; EFS, event-free survival; HRQoL, health-related quality of life; IFN-γ, interferon-gamma; ILND, index lymph node dissection; MPR, major pathologic response; NR, not reported; OS, overall survival; pCR, pathologic complete response; pnCR, pathologic near-complete response; pNR, pathologic nonresponse; RFS, relapse-free survival; T-reg, regulatory T cells; TLND, total lymph node dissection; TMB, tumor mutation burden; trAE, treatment-related adverse events.

^a^ No systemic treatment history.

### Efficacy of Dual Checkpoint Inhibition vs Anti-PD1 Alone

#### Radiologic Response to Treatment

Of 204 patients enrolled in neoadjuvant DCPI arms reporting radiologic assessment results, 21 patients (10.29%) had a radiologic complete response (rCR), 81 patients (39.71%) had a radiologic partial response, and 74 patients (36.27%) had stable disease on radiologic examination. Radiologic progressive disease (rPD), either locally or as distant metastasis, was noted in 24 patients (11.76%). Of 160 patients enrolled in neoadjuvant anti-PD1 trials with reported radiologic examination results, 9 patients (5.63%) had rCR, 64 patients (40%) had a radiologic partial response, and 25 patients (15.63%) had rPD (Table 2).^10,13,14,15,16,17,18^

**Table 2.** Radiologic and Pathologic Patient Outcomes in Neoadjuvant Checkpoint Inhibitor Studies of High-Risk Resectable Melanoma

Study	Patients, No.	Follow-up, median (range), mo	Planned surgical resection, No.	Radiologic response, No.				Pathologic response, No.				Discordance between radiologic and pathologic response
				CR	PR	SD	PD	CR	nCR	PR	>PR	
Amaria et al,^13^ 2018												
Nivolumab	12	15.0 (5.8-22.6)	10	0	3	5	2	3	NR	NR	NR	Yes
Ipilimumab + nivolumab	11	15.6 (5.8-24.4)	11	1	7	2	1	5	NR	NR	NR	Yes
Blank et al,^10^ 2018												
NAT arm^a^	10	21.6 (15.9- NR)	10	0	4	3	1	3	3	1	2	Yes
Adjuvant arm	10	NR										
Huang et al,^14^ 2019												
NAT trial	29	25.0	29	0	3^b^	3^b^	NR	5	3	15	NR	No
Rozeman et al,^15^ 2019												
Group 1	30	24.6 (21.6-27.6)	30	2	17	9	2	14	7	3	6	Yes
Group 2	30		29	3	14	10	2	17	2	4	7	Yes
Group 3	26		24	1	8	12	5	6	6	5	8	Yes
Amaria et al,^16^ 2022												
Single NAT arm	30	24.4 (7.1-34.6)	29	0	18	9	3	17	2	2	8	Yes
Reijers et al,^17^ 2022												
Single NAT arm	99	28.1 (25.0-33.8)	90	14	31	38	13	48	12	11	19	Yes
Patel et al,^18^ 2023												
NAT-adjuvant arm	144^c^	14.7	127	9	58	54	23	28^d^	NR	NR	NR	Yes
Adjuvant only arm	151		151	NR	NR	NR	NR	NR	NR	NR	NR	NR

Abbreviations: CR, complete response; nCR, near-complete response; NAT, neoadjuvant therapy; NR, not reported; PD, progressive disease; PR, partial response; SD, stable disease.

^a^ In the neoadjuvant arm, radiologic data were available from 8 patients, and pathologic data from 9 patients.

^b^ Pathologic response was assessed in 27 patients, and radiologic response was assessed in 6 patients.

^c^ Three patients either withdrew consent or defected. All statistics are done with 141 as the baseline number.

^d^ Among 132 patients evaluated.

Although the rCR was numerically higher in the trials with DCPI arms, no statistically significant difference between the odds of obtaining rCR (OR, 1.93 [95% CI, 0.86-4.33]; *P* = .11) or a radiologic overall objective response (rOOR) (OR, 1.19 [95% CI, 0.79-1.81]; P=.41) was observed between the 2 groups. Likewise, the results were not statistically significant despite fewer patients having had rPD in the DCPI group compared with anti-PD1 monotherapy (OR, 0.72 [95% CI, 0.39-1.32]; *P* = .29) (Table 3).

**Table 3.** Outcomes Comparing Dual Immune Checkpoint Inhibitor With Single-Agent Anti-PD1 in the Neoadjuvant Setting

Variable	No. (%)		OR (95% CI)	*P* value
	DCPI	Anti-PD1		
Total patients	206	182	NA	NA
Radiologic response evaluated	204	160	NA	NA
rCR	21 (10.3)	9 (5.6)	1.93 (0.86-4.33)	.11
rOOR	102 (50.0)	73 (45.6)	1.19 (0.79-1.81)	.41
rPD	24 (11.8)	25 (15.6)	0.72 (0.39-1.32)	.29
Pathologic response evaluated	205	173	NA	NA
pCR	93 (45.4)	36 (22.5)	3.16 (1.99-4.99)	.001
Adverse events				
Grade 3 or 4 irAE	70 (33.9)	22 (12.1)	3.75 (2.20-6.37)	.001
Unable to complete NAT	95 (46.1)	14 (7.7)	10.27 (5.58-18.91)	.001
Planned surgical resection	194 (94.2)	166 (91.2)	1.56 (0.72-3.39)	.26

Abbreviations: DCPI, dual checkpoint inhibition; irAE, immune-related adverse event; NA, not applicable; NAT, neoadjuvant immunotherapy; OR, odds ratio; pCR, pathologic complete response; PD1, programmed cell death protein-1; rCR, radiologic complete response; rOOR, radiologic overall objective response; rPD, radiologic progressive disease.

#### Pathologic Response to Treatment

Among 205 patients enrolled in DCPI trials reporting pathologic assessment results, 93 patients (45.36%) treated with neoadjuvant DCPI, and 36 of 173 patients (20.81%) treated with neoadjuvant anti-PD1 monotherapy achieved a pathologic complete response (pCR). Neoadjuvant DCPI was significantly associated with higher odds of achieving pCR (OR, 3.16 [95% CI, 1.99-4.99]; *P* < .001) (Table 3).

### Subgroup Analysis

#### Conventional-Dose Ipilimumab and Nivolumab vs Alternative-Dose Ipilimumab and Nivolumab

In the neoadjuvant arms of the selected trials, 51 patients received the conventional-dose IPI-NIVO, and 129 patients received the alternative-dose IPI-NIVO. Among the recipients of the conventional-dose IPI-NIVO arm, radiologic response was reported in 49 patients, and pathologic outcomes were reported in 50 patients. Radiologic and pathologic data were available for 129 patients enrolled in the alternative-dose IPI-NIVO arms. No statistically significant difference was found between the 2 regimens regarding rCR, rOOR, rPD, and pCR (Table 4).

**Table 4.** Conventional-Dose Ipilimumab and Nivolumab vs Alternative-Dose Ipilimumab and Nivolumab in the Neoadjuvant Setting

Variable	Dose, No. (%)		OR (95% CI)	*P* value
	Conventional dose^a^	Alternative dose^b^		
Total patients	51	129	NA	NA
Radiologic response evaluated	49	129	NA	NA
rCR	3 (6.1)	17 (13.2)	0.43 (0.12-1.54)	.19
rOOR	31 (63.3)	62 (48.1)	1.86 (0.95-3.66)	.07
rPD	4 (8.2)	15 (11.6)	0.68 (0.21-2.15)	.51
Pathologic response evaluated	50	129	NA	NA
pCR	22 (44.0)	65 (50.4)	0.77 (0.40-1.49)	.44
Adverse events				
Grade 3 or 4 irAE	29 (56.9)	28 (21.7)	4.76 (2.38-9.52)	.001
Unable to complete NAT	13 (26.0)	15 (11.6)	2.6 (1.14-5.95)	.02
Planned surgical resection	51 (100)	119 (92.2)	9.05 (0.52-157.37)	.13

Abbreviations: irAE, immune-related adverse event; NA, not applicable; NAT, neoadjuvant therapy; OR, odds ratio; pCR, pathologic complete response; rCR, radiologic complete response; rOOR, radiologic overall objective response; rPD, radiologic progressive disease.

^a^ Conventional dose: 3-mg/kg dose of ipilimumab + 1-mg/kg dose of nivolumab.

^b^ Alternative dose: 1-mg/kg dose of ipilimumab + 3-mg/kg dose of nivolumab.

#### Anti-PD1 vs Alternative-Dose Ipilimumab and Nivolumab

A total of 182 patients were treated with neoadjuvant anti-PD1 monotherapy. The radiologic response was reported in 160 patients, and the pathologic response was reported in 173 patients. When comparing the 129 patients receiving the alternative-dose IPI-NIVO regimen, this dosage was significantly associated with increased odds of achieving rCR (OR, 2.55 [95% CI, 1.10-5.93]; *P* = .03) and pCR (OR, 3.87 [95% CI, 2.34-6.40]; *P* < .001) compared with anti-PD1 monotherapy (eTable 2 in Supplement 1).

#### Anti-PD1 vs Conventional-Dose Ipilimumab and Nivolumab

The conventional-dose IPI-NIVO regimen was associated with increased odds of achieving an rOOR compared with anti-PD1 monotherapy (OR, 1.95 [95% CI, 1.01-3.77]; *P* = .046). Likewise, conventional-dose IPI-NIVO was associated with greater odds of achieving pCR (OR, 2.99 [95% CI, 1.53-5.83]; *P* < .001) (eTable 3 in Supplement 1).

### Surgical Resection

Of 388 evaluable patients enrolled in the NAT arms, 360 (92.78%) underwent the planned surgical resection. Of 182 evaluable patients enrolled in the anti-PD1 arms, 166 (91.21%) underwent the planned surgical resection. Of 206 patients enrolled in the neoadjuvant DCPI arms, 194 (94.17%) underwent the planned surgical resection. No statistically significant difference was found between the 2 groups’ odds of not undergoing planned surgical resection (DCPI vs anti-PD1 monotherapy: OR, 1.56 [95% CI, 0.72-3.39]; *P* = .26). Comparing conventional-dose IPI-NIVO with alternative-dose IPI-NIVO, no statistically significant difference was observed in patients undergoing surgical resection (Table 3). Similarly, no statistically significant difference was noted when comparing anti-PD1 monotherapy with alternative-dose IPI-NIVO or conventional-dose IPI-NIVO (eTables 2 and 3 in Supplement 1).

### Adverse Events

#### Trials With Anti-PD1 Alone vs Anti-PD1 and Anti-CTLA-4 Combination

Of 388 evaluable patients enrolled in the NAT arms, 338 patients (87.11%) received all planned NAT doses (eTable 4 in Supplement 1). Of 182 evaluable patients in the anti-PD1 monotherapy arm, 168 (92.31%) received all planned doses of NAT. Of 206 patients in the anti-PD1 and anti-CTLA-4 combination arm, only 111 patients (53.89%) could receive all planned doses of NAT. A significantly higher number of patients in the DCPI arm were not able to receive all the planned doses of NAT compared with those in the anti–PD1 monotherapy arm (OR, 10.27 [95% CI, 5.58-18.91]; *P* < .001). Of 388 patients receiving NAT, 92 (23.71%) experienced grade 3 or 4 irAEs. However, only 22 of 182 evaluable patients (12.09%) receiving anti-PD1 monotherapy as NAT had grade 3 or 4 irAEs, compared with 70 of 206 evaluable patients (33.82%) who received DCPI. DCPI was associated with significantly higher odds of irAEs compared with anti-PD1 monotherapy (OR, 3.75 [95% CI, 2.20-6.37]; *P* < .001) (Table 3). Although irAEs were the most common reason for not receiving the complete course of NAT in DCPI, progressive disease was the most common cause of discontinuing NAT in patients receiving anti-PD1 monotherapy (the majority seen in the S1801 trial^2,18^) (Table 4).

#### Conventional-Dose Ipilimumab and Nivolumab vs Alternative-Dose Ipilimumab and Nivolumab

A total of 29 patients in the conventional-dose IPI-NIVO arm and 28 in the alternative-dose IPI-NIVO arm experienced a grade 3 or 4 irAE while receiving NAT. The conventional-dose IPI-NIVO arm was associated with higher odds of grade 3 or 4 irAEs compared with the alternative-dose IPI-NIVO arm (OR, 4.76 [95% CI, 2.38-9.52]; *P* < .001). Thirteen patients (25.49%) in the conventional-dose IPI-NIVO arm and 15 patients (11.63%) in the alternative-dose IPI-NIVO arm could not complete the entire planned course of NAT. The conventional-dose IPI-NIVO arm was associated with significantly higher odds of a patient being unable to receive the entire course of NAT compared with the alternative-dose IPI-NIVO arm (OR, 2.60 [95% CI, 1.14-5.95]; *P* = .02) (Table 4).

#### Anti-PD1 vs Alternative-Dose Ipilimumab and Nivolumab

A total of 22 patients (12.09%) in the anti-PD1 arm and 28 patients (21.71%) in the alternative-dose IPI-NIVO arm experienced a grade 3 or 4 irAE during the NAT period. Alternative-dose IPI-NIVO was significantly associated with higher odds of grade 3 or 4 irAEs compared with anti-PD1 monotherapy (OR, 2.02 [95% CI, 1.09-3.72]; *P* = .02) (eTable 2 in Supplement 1).

#### Anti-PD1 vs Conventional-Dose Ipilimumab and Nivolumab

Compared with anti-PD1 monotherapy, conventional-dose IPI-NIVO was associated with significantly higher odds of grade 3 or 4 irAEs (29 of 51 patients [56.86%] vs 22 of 182 patients [12.09%]; OR, 9.59 [95% CI, 4.71-19.52]; *P* < .001). A significant association was found between the conventional-dose IPI-NIVO treatment regimen and patients not receiving all planned doses of NAT (13 of 51 patients [25.49%] vs 14 of 182 patients [7.69%]; OR, 4.11 [95% CI, 1.78-9.44]; *P* < .001) (eTable 3 in Supplement 1).

## Discussion

The prospect of achieving a pCR with NAT in patients with melanoma, which may lead to better survival, bolsters a compelling argument for developing and validating neoadjuvant approaches to treating patients with HRRM. The durability of responses to ICIs has been apparent in trials undertaken in metastatic unresectable disease and is likely to be confirmed in HRRM.^19^ The durable relapse-free survival benefit observed in patients who had a pCR with NAT suggests advantages for neoadjuvant ICI treatment in larger phase 2 and 3 trials.^16,20^ On the other hand, when the NAT results in pathologic nonresponse (pNR), it suggests the likelihood of tumor progression within the first 2 years of surgery.^17,20^ Therefore, NAT has been considered an emerging approach for patients with HRRM, although the optimal regimen for NAT remains unclear.

In this pooled analysis, we assessed 3 key components—efficacy of the regimen, ability to undergo surgical resection, and whether grade 3 or 4 irAEs were incurred. These are deciding factors for any neoadjuvant approach. We included surgical resection separately, as surgery is the definitive treatment for HRRM. In the overall analysis, radiologic responses were not significantly different among trials of anti-PD1 and the DCPI interventions. However, DCPI therapy was associated with greater odds of achieving pCR with NAT, despite significantly higher deviations from planned NAT, suggesting greater efficacy of the DCPI regimens. The significantly higher pCR rates remain even when anti-PD1 arms are compared with the conventional-dose IPI-NIVO and the alternative-dose IPI-NIVO arms separately (eTables 2 and 3 in Supplement 1). Although DCPI was associated with greater odds of achieving pCR, DCPI was also associated with increased risk for grade 3 or 4 irAEs. The difference remains significant even when anti-PD1 arms were individually compared with the conventional-dose IPI-NIVO and alternative-dose IPI-NIVO arms, establishing the safety profile of anti-PD1 in NAT.

We conducted a separate subgroup analysis comparing the 2 regimens to understand the difference between the conventional-dose IPI-NIVO and alternative-dose IPI-NIVO regimens. Although there was no significant difference in the odds of achieving a radiologic response or pCR between the alternative-dose IPI-NIVO and the conventional-dose IPI-NIVO, a trend towards significance for improved rOOR with conventional-dose IPI-NIVO and a better rate of pCR with alternative-dose IPI-NIVO emerged during statistical analysis. However, the risk for irAEs was significantly higher with conventional-dose IPI-NIVO treatment, and significantly fewer patients could complete the planned NAT with this regimen. Given the low number of patients in the conventional-dose IPI-NIVO arm, establishing the benefits of conventional-dose IPI-NIVO is challenging. However, this analysis suggests that the alternative-dose IPI-NIVO may afford similar benefits in the neoadjuvant setting with reduced risk for irAEs.

### Future Direction in Neoadjuvant Trials

Although the outcomes of NAT for patients with HRRM and a pathologic response are favorable, the optimal management of patients with PD or pNR remains uncertain. All trials report worse outcomes for such patients, with relapses often within the first 2 years. In the PRADO trial, patients with pNR were offered standard adjuvant systemic therapy with nivolumab, BRAF/MEK inhibitors (for those with *BRAF* V600E or V600K variants), or radiation therapy. Seventeen patients with pNR received adjuvant systemic therapy. Two of 7 in the nivolumab group and 3 of 10 in the BRAF/MEK inhibitor group relapsed within 24 months. In this trial, the 24-month relapse-free survival rate for the nivolumab group was 71%, and for the BRAF/MEK inhibitor group, the relapse-free survival rate was 90%. Two of 3 patients without subsequent standard adjuvant systemic therapy relapsed within 24 months. The outcomes of patients receiving adjuvant therapy were numerically better than in the prior Opacin-NEO trial,^15,20^ in which no adjuvant therapy was offered to patients with pNR. However, substantially larger and more mature trial data are needed before conclusions can be drawn regarding whether postoperative systemic therapy or surgery can be modified based on the NAT results. The foundation for NAT is the hypothesis that administration of immunotherapy before resection of the (nodal) tumor may achieve qualitatively or quantitatively better immunologic outcomes, with the expansion of relevant clonal T-cell populations. This question still needs to be addressed.

Second, a question has arisen as to whether we need to continue standard postoperative adjuvant therapy for patients who achieve a pCR after receiving neoadjuvant immunotherapy. The design of the PRADO trial^17^ will begin to address this question in part by omitting complete lymph node dissection and standard postoperative adjuvant therapy in patients with a major pathologic response (pCR and pathologic near-complete response [pnCR]; eTable 1 in Supplement 1) in the index lymph node. However, the definition of the index node has not been established, and although the respective 24-month distant metastasis-free survival and relapse-free survival rates of 98% and 93% are encouraging, the data from the NADINA trial (NCT04949113)^21^ will also be important to consider. The NADINA trial randomizes patients between 2 groups, in which 1 receives neoadjuvant DCPI for 2 cycles followed by total lymph node dissection (TLND), and the other receives up-front TLND followed by adjuvant therapy. In this trial, patients in the neoadjuvant arm will receive adjuvant therapy only if the resected tumor does not show a pCR or pnCR. This randomized design of the NADINA trial^21^ differs from S1801,^2,18^ in which the pathologic response after NAT did not drive the decision to give adjuvant therapy to patients. Hence, this trial will help establish the durability of improved clinical outcomes and the need for adjuvant therapy in patients achieving pCR or pnCR.

Third, the radiologic response assessment grossly underestimates the response observed in the pathologic assessment. Table 2^10,13,14,15,16,17,18^ lists the discordance between the radiologic responses and pathologic responses. In the neoadjuvant relatlimab-nivolumab trial, 1 patient with rPD and 3 with stable disease on radiologic examination achieved a major pathologic response. Whether these read-outs reveal the curative mechanisms of NAT, once illuminated through translational immunologic analyses, remains uncertain. Radiomics-based approaches are being developed in metastatic melanoma, which may play a larger role in patients with HRRM receiving NAT and determine who will optimally benefit from surgical resection.^22^ Last, the underrepresentation of patients with mucosal, uveal, and acral lentiginous melanoma in the reported trials to date raises questions about the interpretation of NAT results and potential benefits for patients with these melanoma subtypes. These rarer subtypes of melanoma are known to have poorer responses to checkpoint inhibition, and the benefits of NAT in these subsets may be more challenging than for cutaneous melanoma.

### Limitations

The data for this pooled analysis were derived from the text of the published articles and data presented in references. We have also not adjusted for inherent heterogeneity among studies due to the lack of access to individual patient data. Hence, some associations observed here may be overestimated or underestimated. We have diligently collected data from the articles and provided data in the supplements to maximize accuracy. We also omitted analysis of outcomes in which complete data was not presented. The second limitation of this analysis lies in the inherent heterogeneity of study designs and reporting data. Many clinical variables were not consistently reported across studies, which limits the analysis of the chosen subsets. For instance, we could not find consistent reporting in the adjuvant arms, preventing a detailed analysis of adjuvant therapy vs neoadjuvant therapy. However, in the neoadjuvant arms, the heterogeneity in designs and data reporting was more limited, allowing for analysis of outcomes of a neoadjuvant approach.

## Conclusions

In this pooled analysis of 6 clinical trials, neoadjuvant ICI strategies show sustained clinical responses, particularly in achieving pCR. We found that DCPI-based regimens were associated with higher pCR odds than anti-PD1 monotherapy; however, they also were associated with increased risk for grade 3 or 4 irAEs. Within neoadjuvant DCPI (nivolumab and ipilimumab), the alternative-dose regimen was associated with a better adverse event profile than the conventional-dose regimen, with no significant efficacy differences. Translational immunologic assessments of NAT are needed, and future investigations comparing immunologic outcomes of relatlimab-nivolumab combinations with IPI-NIVO will be pivotal. While acknowledging the heterogeneity of neoadjuvant treatment arms, these findings have provided a foundation for informed discussions between physicians, allied health professionals, and patients with HRRM regarding neoadjuvant ICI regimens available for their consideration. The need for randomized clinical trials with uniform reporting will be necessary to validate these results in the future.

## Notes

Supplement 1. **eTable 1.** Definitions of radiologic and pathologic responses as listed in the studies evaluating neoadjuvant checkpoint inhibitors in melanoma **eTable 2.** Outcomes comparing alternative dose of ipilimumab and nivolumab vs anti-PD1 monotherapy in the neoadjuvant setting **eTable 3.** Outcomes comparing conventional dose of ipilimumab and nivolumab vs anti-PD1 monotherapy in the neoadjuvant setting **eTable 4.** Adverse events reports in the neoadjuvant checkpoint inhibitor trials in melanoma **eMethods.** Supplemental methods
eReferences

Supplement 2. Data Sharing Statement
