# Supplementary material for: Neoadjuvant Dual Checkpoint Inhibitors vs Anti-PD1 Therapy in High-Risk Resectable Melanoma: A Pooled Analysis
Source: JAMA Oncol. 2024 Mar 28;10(5):612–20. doi: 10.1001/jamaoncol.2023.7333 (PMC10979364; doi:10.1001/jamaoncol.2023.7333)
Supplement: Supplement 1. — eTable 1. Definitions of radiologic and pathologic responses as listed in the studies evaluating neoadjuvant checkpoint inhibitors in melanoma eTable 2. Outcomes comparing alternative dose of ipilimumab and nivolumab vs anti-PD1 monotherapy in the neoadjuvant setting eTable 3. Outcomes comparing conventional dose of ipilimumab and nivolumab vs anti-PD1 monotherapy in the neoadjuvant setting eTable 4. Adverse events reports in the neoadjuvant checkpoint inhibitor trials in melanoma eMethods. Supplemental methods eReferences [file jamaoncol-e237333-s001.pdf]

Supplemental Online Content

Mangla A, Lee CM, Mirsky MM, et al. Neoadjuvant dual checkpoint inhibitors vs anti-PD1 therapy in high-risk resectable melanoma: a pooled analysis. *JAMA Oncol*. Published March 28, 2024. doi:10.1001/jamaoncol.2023.7333

eTable 1. Definitions of radiologic and pathologic responses as listed in the studies evaluating neoadjuvant checkpoint inhibitors in melanoma

eTable 2. Outcomes comparing alternative dose of ipilimumab and nivolumab vs anti-PD1 monotherapy in the neoadjuvant setting

eTable 3. Outcomes comparing conventional dose of ipilimumab and nivolumab vs anti-PD1 monotherapy in the neoadjuvant setting

eTable 4. Adverse events reports in the neoadjuvant checkpoint inhibitor trials in melanoma

eMethods. Supplemental methods

eReferences

This supplemental material has been provided by the authors to give readers additional information about their work.

© 2024 Mangla A et al. *JAMA Oncology.*

Quantitative measure	Definition
Pathologic Responses	
Pathologic Complete Response (pCR)	No viable tumor seen on pathologic examination
Near Complete pathologic response (pNCR)	Less than 10% of viable tumor seen on pathologic examination
Partial response (pPR)	More than 10% but less than 50% of viable tumor see on pathologic exam
More than partial response (>pPR)	More than 50% of viable tumor seen on pathologic exam
Radiologic Response (used RECIST 1.1 criteria)	
Complete Response	Complete resolution of target lesion on radiologic exam
Partial Response	More than 50% reduction of target lesion on radiologic exam
Stable Disease	Less than 50% resolution or less than 20% growth on radiologic exam
Progressive disease	More than 20% growth on radiologic exam

eTable 1. Definitions of radiologic and pathologic responses as listed in the studies evaluating neoadjuvant checkpoint inhibitors in melanoma

© 2024 Mangla A et al. *JAMA Oncology.*

	Alternative Dose*	Anti-PD1**	Odds ratio	95% CI	P-value
Total patients	129	182			
Radiologic Response Evaluated	129	160			
rCR	17 (13.2)	9 (5.6)	2.55	1.1 to 5.92	**0.03**
rOOR	62 (48.1)	75 (46.9)	1.05	0.66 to 1.67	0.84
rPD	15 (11.6)	25 (15.6)	0.71	0.36 to 1.41	0.33
Pathologic slides available	129	173			
pCR	65 (50.4)	36 (20.8)	3.87	2.34 to 6.4	**<0.01**
Adverse Events					
Grade 3 or 4 irAE	28 (21.7)	22 (13.8)	2.02	1.09 to 3.72	**<0.02**
Unable to complete NAT	15 (11.6)	14 (8.8)	1.58	0.73 to 3.39	0.24
Planned surgical resection	119 (92.3)	166 (91.2)	0.87	0.38 to 1.99	0.74

eTable 2. Outcomes comparing alternative dose of ipilimumab and nivolumab vs anti-PD1 monotherapy in the neoadjuvant setting

CI- Confidence Interval; irAE- Immunotherapy-related Adverse Event; NAT- Neoadjuvant therapy; rOOR- Radiologic Overall Objective Response; pCR-Pathologic Complete Response; PD-1- Programmed cell death protein-1; rCR- Radiologic Complete Response; rPD- Radiologic Progressive Disease.

* Alternative Dose- Ipilimumab 1mg/kg + Nivolumab 3mg/kg

**Anti-PD1- Includes both pembrolizumab and nivolumab as monotherapy.

	Conventional Dose* (%)	Anti-PD1** (%)	Odds ratio	95% CI	*P*-value

© 2024 Mangla A et al. *JAMA Oncology.*

Total patients	51	182			
Radiologic Response Evaluated	49	160			
rCR	3 (6.1)	9 (5.6)	1.09	0.28 to 4.21	0.9
rOOR	31 (63.3)	75 (46.9)	1.95	1.01 to 3.77	**0.046**
rPD	4 (8.2)	25 (15.6)	0.48	0.16 to 1.45	0.19
Pathologic slides available	50	173			
pCR	22 (44)	36 (20.8)	2.99	1.53 to 5.83	**<0.01**
Adverse Events					
Grade 3 or 4 irAE	29 (56.9)	22 (12.3)	9.59	4.71 to 19.52	**<0.01**
Unable to complete NAT	13 (25.5)	14 (7.7)	4.11	1.78 to 9.44	**<0.01**
Planned surgical resection	51 (100)	166 (91.2)	0.1	0.01 to 1.66	0.11

eTable 3. Outcomes comparing conventional dose of ipilimumab and nivolumab vs anti-PD1 monotherapy in the neoadjuvant setting

CI- Confidence Interval; irAE- Immunotherapy-related Adverse Event; NAT- Neoadjuvant therapy; rOOR- Radiologic Overall Objective Response; pCR-Pathologic Complete Response; PD-1- Programmed cell death protein-1; rCR- Radiologic Complete Response; rPD- Radiologic Progressive Disease.

* Conventional Dose- Ipilimumab 3mg/kg + Nivolumab 1mg/kg

**Anti-PD1- Includes both pembrolizumab and nivolumab as monotherapy.

© 2024 Mangla A et al. *JAMA Oncology.*

	Number of patients	Patients receiving all NAT doses	Reason for NAT discontinuation	Patients completing the entire planned length of treatment	Patients receiving planned resection	Grade 3/4 AE during NAT	Most common Grade 3 AE during NAT	Grade 3 or 4 AE during adjuvant therapy	Most common Grade 3 AE during adjuvant therapy
Amaria et al. 2018									
Nivolumab	12	12	--	--	10	1 (8%)	Tumor pain	2 (20%)	Colitis<br>DKA
Ipilimumab + Nivolumab	11	4		--	11	8 (73%)	Transaminitis<br>Colitis<br>Pneumonia	2 (18%)	Hypophysitis
Blank et al. 2018									
NAT arm	10	8	NR	1	10	9 (90%)	Colitis<br>Elevated Lipase<br>Dermatitis/ Rash<br>Transaminitis	--	--
Adjuvant arm	10	--		1	10	--	--	9 (90%)	Elevated lipase<br>Colitis/ Diarrhea<br>Adrenal insufficiency<br>Transaminitis
Huang et al. 2019									
NAT phase	29	29	--	--	29	3	Rash	NR	NR
Rozeman et al. 2019									
Ipi 3 + Nivo 1	30	26	irAE-4	30	30	12 (40%)	Transaminitis	--	--
Ipi 1 + Nivo 3	30	25	irAE-5	29	29	6 (20%)	Transaminitis	--	--
Ipi 3 & Nivo 3¶	26	18	irAE-8	24	24	13 (50%)	Colitis	--	--

© 2024 Mangla A et al. *JAMA Oncology.*

Amaria et al. 2022									
NAT phase	30	29	PD- 1	--	29	0	--	--	--
Adjuvant Phase	27	--		18	--	--	--	7 (26%)	Adrenal Insufficiency<br>Transaminitis<br>Arthralgia<br>Hyponatermia
Reijers et al. 2022									
NAT arm†	99	89	- PD- 6<br>- irAE- 3	89	90	22 (22%)	Transaminitis<br>Colitis and diarrhea<br>Rash	--	--
Patel 2023 (As per data available at the time of publication)									
NAT-adjuvant arm	144*	127	- PD- 12<br>- irAE- 1<br>- Consent withdraw- 2<br>- Other-1	50**	127*	18 (12%)	Transaminitis	--	--
Adjuvant only arm	151*	--		38**	151*	--	--	22 (14%)	Maculopapular Rash<br>Pruritis<br>Vomiting

eTable 4. Adverse events reports in the neoadjuvant checkpoint inhibitor trials in melanoma

AE- Adverse Event; CK- Creatinine Kinase; DKA- Diabetic Ketoacidosis; Ipi- Ipilimumab, irAE- Immune Related Adverse Event; Nivo- Nivolumab; NAT- Neoadjuvant Therapy; NR- Not reported; PD- Progressive Disease

Group C was closed prematurely due to severe Grade 3-4 AE in this group.

† Adjuvant therapy was not standardized in patients.

*Data of patients available at the time of analysis. Three patients withdrew consent or defected, hence only 141 patients used for statical analysis.

**At the time of data cut-off these patients completed the protocol specified goals. In the NAT-arm, 14 patients in the NAT-adjuvant arm and 21 patients in the adjuvant-only arm did not receive adjuvant therapy after surgery.

© 2024 Mangla A et al. *JAMA Oncology.*

eMethods

The odds ratio (OR), its standard error and 95% confidence interval are calculated according to Altman, 1991.

1. The odds ratio is given by:

$$
\begin{aligned}
OR &= \frac{a/b}{c/d} \\
&= \frac{a \times d}{b \times c}
\end{aligned}
$$

	Positive outcome	Negative outcome
Exposed group	a	b
Non-Exposed group	c	d

2. Standard error of the log odds ratio

$$\mathrm{SE}\{\ln(OR)\} = \sqrt{\frac{1}{a} + \frac{1}{b} + \frac{1}{c} + \frac{1}{d}}$$

3. 95% confidence interval is calculated using this equation:

$$95\%\ \mathrm{CI} = \exp\Big(\ \ln(OR) - 1.96 \times \mathrm{SE}\{\ln(OR)\}\ \Big) \quad \text{to} \quad \exp\Big(\ \ln(OR) + 1.96 \times \mathrm{SE}\{\ln(OR)\}\ \Big)$$

© 2024 Mangla A et al. *JAMA Oncology.*

Where zeros cause problems with computation of the odds ratio or its standard error, 0.5 is added to all cells (a, b, c, d) (Pagano & Gauvreau, 2000; Deeks & Higgins, 2010).

Test of significance: the P-value is calculated according to Sheskin, 2004 (p. 542). A standard normal deviate (*z*-value) is calculated as ln(OR)/SE{ln(OR)}, and the P-value is the area of the normal distribution that falls outside ±*z*

© 2024 Mangla A et al. *JAMA Oncology.*

eReferences:

1. Altman DG (1991) Practical statistics for medical research. London: Chapman and Hall.
2. Altman DG, Deeks JJ, Sackett DL. Odds ratios should be avoided when events are common [letter]. BMJ 1998;317:1318.
3. Deeks JJ, Higgins JPT (2010) Statistical algorithms in Review Manager 5. Retrieved from https://training.cochrane.org/
4. Kirkwood BR, Sterne JAC (2003) Essential medical statistics, 2nd ed. Oxford: Blackwell Science.
5. Pagano M, Gauvreau K (2000) Principles of biostatistics. 2nd ed. Belmont, CA: Brooks/Cole.
6. Parshall MB (2013) Unpacking the 2 x 2 table. Heart & Lung 42:221-226.
7. Sheskin DJ (2004) Handbook of parametric and nonparametric statistical procedures. 3rd ed. Boca Raton: Chapman & Hall /CRC.

© 2024 Mangla A et al. *JAMA Oncology.*
