# Supplementary material for: Neoadjuvant Dual Checkpoint Inhibitors vs Anti-PD1 Therapy in High-Risk Resectable Melanoma: A Pooled Analysis
Source: JAMA Oncol. 2024 Mar 28;10(5):612–20. doi: 10.1001/jamaoncol.2023.7333 (PMC10979364; doi:10.1001/jamaoncol.2023.7333)
Supplement: Supplement 2. — Data Sharing Statement [file jamaoncol-e237333-s002.pdf]

Data Sharing Statement

Mangla. Efficacy and Safety of Neoadjuvant Dual Checkpoint Inhibition in High-Risk Resectable Melanoma. *JAMA Oncol*. Published March 28, 2024.
doi:10.1001/jamaoncol.2023.7333

Data
Data available: No
